# Pregnancy Outcomes in Women With Thalassemia Trait: A Multicenter Cohort Study

**DOI:** 10.1155/anem/8899690

**Published:** 2025-12-29

**Authors:** Daria Chelysheva, Ameera Syed, Hannah Ruby, Anna Homeniuk, Anas Atrash, Feras Al Moussally

**Affiliations:** ^1^ Department of Medicine, UPMC Harrisburg, Harrisburg, Pennsylvania, USA; ^2^ Drexel University College of Medicine, Philadelphia, Pennsylvania, USA, drexel.edu; ^3^ Department of Hematology/Oncology, UPMC Harrisburg, Harrisburg, Pennsylvania, USA

## Abstract

**Introduction:**

Thalassemia trait generally has minimal clinical impact, but physiologic changes during pregnancy may increase the risk of anemia, transfusion requirements, and hypertensive disorders. Existing evidence on pregnancy outcomes in this population is limited, with some conflicting data. This study aims to evaluate pregnancy‐related outcomes in patients with thalassemia trait using a large, multicenter database.

**Methods:**

For this retrospective cohort study, we used data from the TriNetX US Collaborative Network. Females aged 18–45 with ICD‐10 codes indicating pregnancy (Z33.1, O00–O9A, Z34, or Z3A) were included. Patients with pregnancy and coexisting thalassemia trait (D56.3) were assigned to the thalassemia cohort (*n* = 22,913), while those without any thalassemia diagnosis comprised the nonthalassemia cohort (*n* = 5,611,147). Propensity score matching was performed to balance age, race/ethnicity, obesity, smoking status, essential hypertension, and Type 2 diabetes mellitus. After 1:1 matching, 22,770 patients remained in each cohort (total *N* = 45,540). Outcomes were assessed within 1 year of the index date, including anemia during pregnancy, blood transfusion, preeclampsia/eclampsia, cesarean delivery, venous thromboembolism (VTE), heart failure/cardiomyopathy, preterm delivery, intrauterine growth restriction (IUGR), and intrauterine fetal demise (IUFD). Risk ratios (RRs) with 95% confidence intervals (CIs) were calculated.

**Results:**

Thalassemia trait was associated with higher risks of anemia during pregnancy (RR: 3.00 and 95% CI: 2.87–3.13), needing blood transfusion (RR: 1.90 and 95% CI: 1.69–2.20), preeclampsia/eclampsia (RR: 1.54 and 95% CI: 1.47–1.61), cesarean delivery (RR: 1.43 and 95% CI: 1.36–1.51), preterm delivery < 37 weeks (RR: 1.40 and 95% CI: 1.31–1.65), and IUGR (RR: 1.96 and 95% CI: 1.72–2.23), all were statistically significant with a *p* < 0.001. Increased risk was also observed for VTE (RR: 1.57 and 95% CI: 1.12–2.20, *p* < 0.001) and IUFD (RR: 1.37 and 95% CI: 1.087–1.75, *p* < 0.001). No significant association was found with heart failure/cardiomyopathy (RR: 1.28 and 95% CI: 0.93–1.76, *p* = 0.124).

**Conclusion:**

Thalassemia trait in pregnancy was associated with increased rates of anemia, transfusion, and adverse maternal and fetal outcomes. Such adverse outcomes include pre‐eclampsia/eclampsia, cesarean delivery, preterm birth and IUGR. These findings underscore the need for tailored peripartum care strategies in this high‐risk population.

## 1. Introduction

Normal hemoglobin is a tetramer composed of two α‐ and two β‐globin chains. Mutations affecting the production of these globin chains lead to thalassemia, one of the most common monogenic disorders worldwide. Thalassemia is broadly classified into α‐ and β‐thalassemia, depending on the affected globin gene. Carriers of thalassemia trait typically have clinically mild disease, often presenting only with microcytosis, mild anemia, and no significant symptoms in nonpregnant individuals [1]. The global prevalence of thalassemia remains highest in the Mediterranean region, the Middle East, and Southeast Asia [2]. A 2025 study in Baghdad reported that 3.5% of the individuals carried β‐thalassemia traits and 0.01% carried δβ‐thalassemia traits [3].

During pregnancy, physiological changes, including approximately 30% expansion of red cell mass and 40% increase in blood volume [4], can exacerbate anemia in women with thalassemia trait. Anemia during pregnancy is commonly defined as hemoglobin < 11 g/dL in the first and third trimesters and < 10.5 g/dL in the second trimester, per American College of Obstetricians and Gynecologists (ACOG) criteria [5]. Data from large cohort studies in China suggest that thalassemia trait may be associated with adverse pregnancy outcomes, including higher rates of hypertensive disorders, cesarean delivery, and preterm birth compared with women without thalassemia [6]. In contrast, other studies report no significant differences in outcomes compared with normal pregnancies [7, 8].

Given these conflicting findings and the generally small sample sizes of prior reports, large‐scale data are needed to clarify the obstetric risks, if any, associated with thalassemia trait. In this study, we leverage a multicenter database to investigate pregnancy outcomes in women with thalassemia trait compared with unaffected women, hypothesizing that even trait‐level thalassemia is associated with higher rates of complications such as anemia and hypertensive disorders of pregnancy.

## 2. Methods

This retrospective, multicenter cohort study used the TriNetX US multicenter electronic health‐record network (https://www.trinetx.com), which aggregates deidentified data from electronic health records, pharmacy records, and administrative billing systems contributed by participating healthcare organizations (HCOs). Compiled data are standardized across HCOs using International Classification of Diseases, Tenth Revision (ICD‐10), and Current Procedural Terminology (CPT) codes. Because only deidentified aggregate data analysis was conducted, this study was exempt from institutional review board oversight.

Female patients aged 18–45 years with ICD‐10 codes indicating pregnancy (O00–O9A, Z33.1, Z34.0, or Z3A) were eligible for inclusion.

Cohort 1 (Thalassemia) consisted of patients with a diagnosis of thalassemia minor (D56.3), excluding those with alpha‐thalassemia (D56.0), beta‐thalassemia major/intermedia (D56.1), other thalassemia (D56.8), or thalassemia unspecified (D56.9). Cohort 2 (Control) included pregnant patients without any thalassemia diagnosis (D56) (Table 1).

**Table 1:** ICD‐10 and CPT codes for cohort build.

Item	Codes
Cohort identification	
Pregnant state, incidental	Z33.1
Pregnancy, childbirth, and the puerperium	O00–O9A
Encounter for supervision of normal pregnancy	Z34.0
Weeks of gestation	Z3A
Thalassemia minor	D56.3
Excluded: alpha thalassemia	D56.0
Excluded: beta thalassemia	D56.1
Excluded: other thalassemias	D56.8
Excluded: thalassemia, unspecified	D56.9
Control group (no thalassemia diagnosis)	D56 (absent)

The index event was defined as the earliest pregnancy‐related encounter meeting inclusion criteria. Outcomes were assessed during a 1‐year time window beginning 1 day after the index event.

Outcomes of interest included anemia during pregnancy, blood transfusion, preeclampsia/eclampsia, cesarean delivery, venous thromboembolism (VTE), heart failure/cardiomyopathy, preterm delivery, intrauterine growth restriction (IUGR), and intrauterine fetal demise (IUFD) (Table 2).

**Table 2:** Maternal and perinatal outcomes and corresponding ICD‐10, CPT, and SNOMED codes.

Outcome	Codes
Anemia during pregnancy	O99.01 (anemia complicating pregnancy), O99.011–O99.013 (trimester specific), D64.9 (anemia unspecified), D50–D53, D55–D59, D60– D64
Blood transfusion	ICD‐10‐PCS 302, CPT 36430, CPT 30233N1, SNOMED 116859006 (blood product transfusion), 116863004 (RBC transfusion), 54790000 (blood component transfusion)
Preeclampsia/eclampsia	O14 (pre‐eclampsia), O14.00 (mild/moderate), O14.10 (severe), O15 (eclampsia), O15.0 (eclampsia complicating pregnancy), O10–O16 (hypertensive disorders in pregnancy)
Cesarean section	O82, CPT 59510, CPT 1014218, CPT 1008991 (cesarean delivery procedures), SNOMED 11466000 (cesarean section)
Venous thromboembolism (DVT/PE)	I26 (pulmonary embolism), I82.40 (DVT of lower extremity), I82.629 (DVT of upper extremity)
Heart failure/cardiomyopathy	I50 (heart failure), I42 (cardiomyopathy)
Preterm delivery	O60.1 (preterm labor with preterm delivery)
Intrauterine growth restriction (IUGR)	O36.5990 (maternal care for poor fetal growth, unspecified trimester)
Intrauterine fetal demise (IUFD)	O36.4 (maternal care for intrauterine death)

Anemia during pregnancy was identified using pregnancy‐specific ICD‐10 codes (O99.0x) in combination with anemia‐related codes (D50–D64). The pregnancy‐specific O99.0x codes are known to be underutilized in routine EHR documentation, particularly across multisite networks such as TriNetX; therefore, the combined coding strategy was used to enhance capture of clinically recognized anemia during pregnancy while maintaining reproducibility.

**Figure 1:**
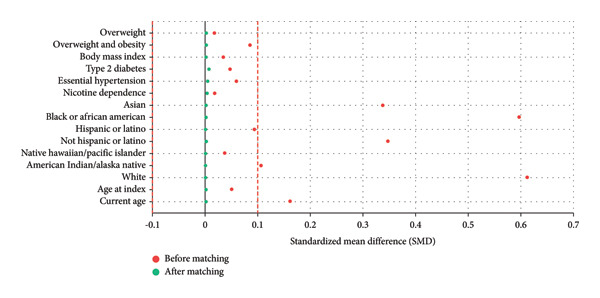
Love plot of covariate balance pre‐ and postmatching.

Propensity score matching (PSM) was performed using a 1:1 nearest‐neighbor algorithm without replacement. Variables included in the matching model were demographics (age, age at index, and race/ethnicity) and comorbidities (body mass index, smoking status, essential hypertension, and Type 2 diabetes mellitus) as shown in Figure 1. After matching, 22,770 patients were included in each cohort (Table 3).

**Table 3:** Baseline demographic and clinical characteristics.

Characteristic	Thalassemia before matching = 22,911	Control before matching = 5,610,874	Thalassemia after matching = 22,770	Control after matching = 22,770
Age at index, mean ± SD	28.1 ± 6.01	27.8 ± 5.87	28.1 ± 6.01	28.1 ± 6.01
Female, *n* (%)	22,911 (100%)	5,610,874 (100%)	22,773 (100%)	22,773 (100%)
Race/ethnicity, *n* (%)				
Not Hispanic or Latino	14,863 (65.3%)	26,439 (48.3%)	14,863 (65.3%)	14,854 (65.2%)
Hispanic or Latino	2391 (10.4%)	740,203 (13.2%)	2391 (10.5%)	2395 (10.5%)
Black or African American	9300 (40.7%)	832,199 (14.8%)	9300 (40.8%)	9314 (40.9%)
White	4487 (19.6%)	2,589,747 (46.2%)	4487 (19.7%)	4494 (19.7%)
Asian	3507 (15.3%)	288,963 (5.1%)	3507 (15.4%)	3493 (15.3%)
Native Hawaiian/Other Pacific Islander	239 (1.0%)	38,705 (0.7%)	239 (1.0%)	240 (1.1%)
American Indian/Alaska Native	382 (1.7%)	30,678 (0.5%)	382 (1.7%)	381 (1.7%)
Comorbidities, *n* (%)				
Nicotine dependence (F17)	272 (1.19%)	76,415 (1.36%)	272 (1.19%)	274 (1.20%)
Hypertension (I10)	325 (1.42%)	44,359 (0.81%)	325 (1.42%)	326 (1.43%)
Type 2 diabetes mellitus (E11)	174 (0.76%)	22,296 (0.40%)	174 (0.76%)	171 (0.75%)
Overweight/obesity (E66/Z68)	671 (2.95%)	91,149 (1.63%)	671 (2.95%)	684 (3.00%)

## 3. Results

A total of 22,913 pregnant patients with thalassemia trait and 5,611,147 without thalassemia were initially identified. After 1:1 PSM, 22,770 women remained in each group (total *N* = 45,540), with balanced baseline characteristics (standardized mean differences < 0.1). The mean age of the matched cohorts was 28.1 years, and distributions of race/ethnicity and comorbidities, including obesity, smoking status, hypertension, and Type 2 diabetes mellitus, were comparable between groups (Table 1).

In the matched analysis, thalassemia trait was associated with significantly higher rates of several adverse maternal and perinatal outcomes compared with controls (Table 2). Anemia during pregnancy occurred in 31.4% of the patients with thalassemia trait versus 10.4% of controls (RR: 3.0 and 95% CI: 2.87–3.13; *p* < 0.001). The need for blood transfusion was also increased in the trait cohort (2.2% vs. 1%; RR: 1.9 and 95% CI: 1.69–2.2; *p* < 0.001).

Hypertensive disorders of pregnancy were more common among patients with thalassemia trait, with preeclampsia/eclampsia diagnosed in 18.9% compared with 12.3% of the controls (RR: 1.54 and 95% CI: 1.47–1.61; *p* < 0.001). Cesarean delivery was more frequent in the trait group (13.4% vs. 9.3%; RR: 1.43 and 95% CI: 1.36–1.51; *p* < 0.001). The risk of preterm birth < 37 weeks was also elevated (3.0% vs. 2%; RR: 1.4 and 95% CI: 1.31–1.65; *p* < 0.001). IUGR occurred in 3.4% of the trait cohort compared with 1.7% of controls (RR: 1.96 and 95% CI: 1.72–2.23; *p* < 0.001).

Less frequent but clinically significant outcomes also demonstrated increased risk. VTE occurred in 0.4% of thalassemia pregnancies compared with 0.2% of controls (RR: 1.57 and 95% CI: 1.12–2.2; *p* < 0.001). IUFD occurred in 0.7% of the trait cohort versus 0.5% of controls (RR: 1.37 and 95% CI: 1.087–1.75; *p* < 0.001). In contrast, heart failure or cardiomyopathy remained rare and did not differ significantly between groups (0.4% vs. 0.3%; RR: 1.28 and 95% CI: 0.93–1.76; *p* = 0.1239) (Tables 4 and 5).

**Table 4:** Standardized mean differences before and after propensity score matching.

Variable	SMD before matching	SMD after matching
Current age	0.161	0.001
Age at index	0.050	0.000
White	0.612	0.001
American Indian/Alaska Native	0.106	0.001
Native Hawaiian/Pacific Islander	0.037	0.001
Not Hispanic or Latino	0.347	0.002
Hispanic or Latino	0.093	0.001
Black or African American	0.596	0.001
Asian	0.337	0.001
Nicotine dependence	0.018	0.004
Essential hypertension	0.059	0.001
Type 2 diabetes mellitus	0.047	0.001
Body mass index	0.034	0.001

**Table 5:** Key pregnancy outcomes in matched cohorts with and without thalassemia trait.

Outcome	Thalassemia trait (*n* = 22,770)	Control (*n* = 22,770)	Relative risk (RR)	95% CI	*p* value
Anemia during pregnancy	7166 (31.4%)	2388 (10.4%)	3.00	2.87–3.13	< 0.001
Blood transfusion	517 (2.2%)	263 (1%)	1.90	1.69–2.20	< 0.001
Preeclampsia/Eclampsia	4320 (18.9%)	2804 (12.3%)	1.54	1.47–1.61	< 0.001
Cesarean delivery	3065 (13.4%)	2134 (9.3%)	1.43	1.36–1.51	< 0.001
Preterm labor (< 37 weeks)	695 (3%)	472 (2%)	1.40	1.31–1.65	< 0.001
Intrauterine growth restriction (IUGR)	789 (3.4%)	401 (1.7%)	1.96	1.72–2.23	< 0.001
Venous thromboembolism (DVT/PE)	88 (0.4%)	56 (0.2%)	1.57	1.12–2.20	< 0.001
Intrauterine fetal demise (IUFD)	160 (0.7%)	116 (0.5%)	1.37	1.087–1.75	< 0.001
Heart failure/cardiomyopathy	86 (0.4%)	67 (0.3%)	1.28	0.93–1.76	0.124

**Figure 2:**
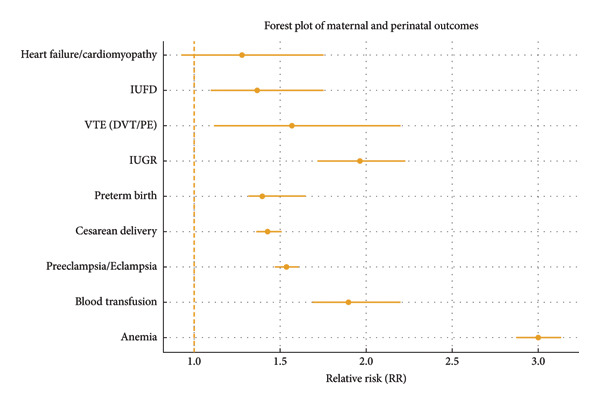
Forest plot: thalassemia trait and pregnancy outcomes.

Overall, thalassemia trait was associated with increased risks of anemia, transfusion, hypertensive disorders of pregnancy, cesarean delivery, preterm birth, fetal growth restriction, and fetal demise, while cardiac complications remained uncommon and statistically similar between groups (Figure 2).

## 4. Discussion

In this multicenter analysis, we found that thalassemia trait is associated with a significantly higher risk of multiple adverse pregnancy outcomes as shown in Figure 2. The most pronounced effect was on maternal hematologic status: nearly one in three thalassemia carriers developed anemia during pregnancy (31.4% vs. 10.4% in controls), reflecting a three‐fold increased risk. This aligns with prior work demonstrating that while carriers are usually asymptomatic, the physiologic stress of pregnancy unmasks their susceptibility to anemia [1]. These results corroborate with prior single‐center studies that reported higher rates of maternal anemia in thalassemia trait pregnancies [9].

Hypertensive disorders of pregnancy were also notably elevated, with preeclampsia/eclampsia occurring in 18.9% of the thalassemia trait pregnancies compared with 12.3% of the controls (RR: 3). This relative increase is consistent with other reports [6, 9]. Several pathways that explain these findings have been proposed in the literature. One such pathway is through oxidative stress since excess free iron and chronic low‐grade hemolysis in thalassemia carriers may increase reactive oxygen species, promoting endothelial dysfunction and hypertension. Another pathway involves iron overload, which, even in carriers, can develop due to unregulated absorption and is linked to vascular and myocardial injury due to prolonged adaptation to chronic anemia [10], which includes resting tachycardia, low blood pressure, enlarged end‐diastolic volume, high ejection fraction, and high cardiac output [7, 11]. Abnormalities of the placenta could also be a part of the process, with studies suggesting that altered trophoblast invasion and impaired uteroplacental blood flow contribute to both preeclampsia and fetal growth restriction [12]. These processes help explain why thalassemia trait may be linked to high blood pressure problems in pregnancy, showing the importance of closer monitoring and early detection of preeclampsia in affected women.

Adverse fetal growth outcomes were another key finding. The rate of IUGR was almost double (3.4% vs. 1.7%, RR: 1.96), likely reflecting the downstream effects of chronic maternal anemia on oxygen delivery to the fetus [12]. Similarly, preterm delivery was more common (3.0% vs. 2%, RR: 1.4). These results were consistent with earlier studies and support the recommendations for enhanced ultrasound surveillance for fetal growth in pregnancies complicated by thalassemia trait [6, 8]. However, not all studies have reported this association; for instance, a Thai hospital–based study did not find a significant difference in birth weight or small for gestational age rates between trait carriers and controls, which could be potentially due to smaller sample size and proactive prenatal interventions [13].

The cesarean delivery rate was notably higher in the trait group (13.4% vs. 9.3%, RR: 1.43). Other studies in Israel and Iran reported similar findings of a higher cesarean rate in β‐thalassemia minor pregnancies but found that thalassemia was not an independent predictor of cesarean delivery after adjusting for factors such as prior cesarean delivery, IUGR, and oligohydramnios [6, 14]. Similarly, in our study, the increase in cesarean delivery may be mediated by the higher incidence of complications rather than an intrinsic need for surgery due to the trait itself. Some prior studies did not find a significant difference in the mode of delivery in thalassemia trait pregnancies [11]. The divergence in our findings could be due to greater power to detect differences, or variations in practice patterns across the diverse hospitals in the database. Regardless, the result signals that thalassemia trait pregnancies more often involve complex deliveries, underlining the importance of delivering in facilities equipped for high‐risk pregnancies if possible.

In our cohort, VTE occurred at a very low absolute rate in both groups, though the relative risk was higher in the thalassemia trait cohort (0.4% vs. 0.2%, RR: 1.57, *p* < 0.001). Given the rarity of events, this finding should be interpreted cautiously and viewed as hypothesis‐generating rather than clinically directive. Although altered erythrocyte membranes and chronic low‐grade hemolysis in thalassemia carriers have been proposed to contribute to a prothrombotic state [15], current evidence and clinical guidelines do not recommend thromboprophylaxis for thalassemia trait in pregnancy in the absence of additional risk factors [5, 16]. Further research is needed to clarify whether specific subgroups of trait carriers may be at meaningfully increased thrombotic risk.

Taken together, these findings support several practical considerations for prenatal care. Although specific guidelines for thalassemia trait are limited, expert consensus emphasizes early and repeated hemoglobin assessment, with evaluation of iron studies prior to supplementation to avoid unnecessary iron overload [1]. Blood pressure monitoring should follow routine schedules, with heightened vigilance for early signs of preeclampsia, given the increased hypertensive risk observed. In addition, serial fetal‐growth ultrasonography may be reasonable, particularly in the third trimester, to monitor for emerging growth restriction. Referral to maternal–fetal medicine may be appropriate for individuals with moderate or worsening anemia, coexisting maternal comorbidities, or development of hypertensive disorders, while hematology consultation may be beneficial when diagnostic uncertainty exists or hemoglobin levels decline despite supplementation. These measures align with general recommendations from international thalassemia care guidelines and national obstetric society statements [1, 5].

## 5. Limitations

This study has several limitations inherent to its retrospective design and use of administrative data. First, identification of thalassemia trait was based on ICD coding in the EHR. This could lead to misclassification; for instance, some women with trait might not have been coded as such (underestimation) or some coded as D56.3 might have a form of thalassemia intermedia or another hemoglobinopathy. Additionally, because the study was conducted using the TriNetX database, coding practices vary across participating health systems, and D56.3 may not consistently distinguish α‐thalassemia trait from β‐thalassemia trait, introducing potential phenotype misclassification.

Second, anemia during pregnancy was identified using both pregnancy‐specific ICD‐10 codes (O99.0x) and broader anemia codes (D50–D64). While this approach captures anemia across a range of clinical documentation styles, the broader D‐series codes may include anemia diagnoses made outside of pregnancy and could, therefore, introduce misclassification. Pregnancy‐specific anemia codes may be underutilized in real‐world EHR data, particularly across large multi‐institution networks such as TriNetX. This limitation may contribute to conservative anemia prevalence estimates and should be considered when interpreting comparisons between groups.

Third, this study lacked additional detailed clinical data such as hemoglobin levels, iron studies, or severity of anemia for each patient. Thus, we could neither distinguish outcomes between women with more severe anemia versus those with normal hemoglobin within the trait group nor assess how well anemia was corrected with supplementation. Similarly, we did not have data on iron supplementation, folate use, or blood transfusion thresholds, which may vary by institution and could influence outcomes like IUGR or preterm birth.

Fourth, although race/ethnicity was included in the PSM, residual confounding related to ancestry is possible. Thalassemia trait prevalence varies by ancestral background and may correlate with unmeasured factors such as socioeconomic status, nutrition, and access to prenatal care, which could influence pregnancy outcomes. Our matching approach mitigates but does not eliminate this source of confounding.

Fifth, our analysis captures outcomes within 1 year of the index; if a patient delivered late and had certain postpartum complications slightly beyond that window, those might be missed. However, most pregnancy‐related outcomes would occur by that timeframe. Our study intentionally focused on maternal carrier status and common obstetric outcomes; we did not examine neonatal outcomes in detail (aside from those implicitly covered by preterm birth and IUGR).

## 6. Conclusion

Pregnancy in women with thalassemia trait is generally well tolerated but is not without increased risk. In this large U.S. cohort, thalassemia trait was associated with a substantially higher incidence of anemia and with elevated risks of preeclampsia/eclampsia, IUGR, preterm delivery, cesarean birth, and need for transfusion. Smaller increases were also observed for VTE and IUFD, though the absolute rates of these outcomes remained low. No excess risk was seen for cardiomyopathy or heart failure. These findings support the need for tailored prenatal and peripartum management in thalassemia carriers, including early anemia screening, close blood pressure surveillance, and serial fetal growth monitoring. Awareness of these risks is the key to timely intervention and counseling, allowing most women with thalassemia trait to anticipate favorable pregnancy outcomes when managed appropriately.

## Ethics Statement

Informed consent for case publication was obtained from the patient. Institutional Review Board (IRB) approval was secured prior to submission.

## Conflicts of Interest

The authors declare no conflicts of interest.

## Author Contributions

Daria Chelysheva, MD: conceptualization; methodology; writing–original draft; writing–review and editing; literature review; and supervision.

Ameera Syed, BS: data curation and investigation.

Hannah Ruby, BS: resources; project administration; and reference management.

Anna Homeniuk, MD: investigation and supporting clinical input.

Anas Atrash, MD: methodology; study design review; and supervision.

Feras Al Moussally, MD: writing–review and editing; critical feedback; and supervision.

## Funding

This research received no funding.

## Data Availability

The data that support the findings of this study are available from the corresponding author upon reasonable request.
